# Causes of Granulomatous Inflammation in Native and Allograft Kidneys: Case Series from A Single Center and A Review of the Literature

**DOI:** 10.5146/tjpath.2021.01561

**Published:** 2022-05-19

**Authors:** Cihan Heybeli, Berna Demir Yuksel, Mehtat Unlu, Mehmet Ası Oktan, Hayri Ustun Arda, Ozcan Uzun, Filiz Yıldırım, Serkan Yıldız, Caner Cavdar, Aykut Sifil, Ali Celik, Sulen Sarıoglu

**Affiliations:** Division of Nephrology, Muş State Hospital, Muş, Turkey; Department of Internal Medicine, Dokuz Eylul University Hospital, Izmir, Turkey; Department of Pathology Dokuz Eylul University Hospital, Izmir, Turkey; Division of Nephrology, Baskent University Hospital, Izmir, Turkey; Dokuz Eylul University Hospital, Izmir, Turkey; Medicana Hospital, Izmir, Turkey

**Keywords:** Granuloma, Renal biopsy, Pathology, Renal

## Abstract

*
Objective:* Granulomatous interstitial nephritis is a rare finding, and etiology differs by geography. We aimed to investigate the distribution of causes of granuloma/granulomata in the kidney and renal survival of these patients in a tertiary care hospital in Western Turkey.

*
Material and Method:* Medical records of adults who underwent a kidney biopsy procedure in our institution between January 2000 and June 2019 were reviewed. Pathology reports were searched for biopsies where a granuloma was identified.

*
Results:* Nineteen of 1121 (1.7%) kidney biopsies included granuloma, 17 in native kidneys, and 2 in transplants. The majority of indications for native kidney biopsy was a rise in serum creatinine. Etiologies of granuloma included the following: pauci-immune vasculitis (n=11, 64.7%), tuberculosis (n=2, 11.8%), drug-induced (n=2, 11.8%), tubulointerstitial nephritis/uveitis (TINU) syndrome (n=1, 5.9%), and systemic-lupus erythematosus (n=1, 5.9%). Despite treatment, 6 of 11 (54.5%) patients with vasculitis developed end-stage kidney disease (ESKD) during the median follow-up of 16 months. Both of the patients with tuberculosis, and the patient with TINU syndrome developed ESKD months after the kidney biopsy, despite appropriate therapies. The only case with drug-induced granuloma and both cases with allograft kidney granuloma responded well to glucocorticoids, achieving a complete renal recovery.

*
Conclusion:* The majority of our series had granuloma in the kidney secondary to vasculitis and renal outcomes appear considerably unfavorable despite treatment, probably related to the primary diagnosis. Multicenter studies are needed to better determine the etiology and outcome of each granuloma etiology at different geographic locations.

## INTRODUCTION

Granulomatous interstitial nephritis (GIN) is a histological diagnosis which comprises less than 1% of all kidney biopsies (1). The primary etiology is wide an includes various systemic inflammatory disorders such as granulomatous polyangitis (GPA), sarcoidosis (2–5), Crohn’s disease (6), Sjögren syndrome (7), malignancies such as chronic lymphocytic lymphoma (8,9), fungal infections (10,11), and mycobacterial infections (12,13). Numerous drugs may also cause GIN and these include diuretics (14), proton-pump inhibitors (15,16), non-steroidal anti-inflammatory drugs (17,18), zoledronic acid (19), captopril (20), ciprofloxacin (21), vancomycin (22), anti-TNF agents (23,24), tramadol (25), atazanavir (26,27), and immunotherapy with ipilimumab and nivolumab (28).

It is not straightforward to differentiate between these causes using renal histology alone, and the diagnosis is usually made based on the clinical presentation and extrarenal findings (29,30). The most common cause differs between geographical regions. Drug-induced GIN and renal involvement of sarcoidosis are quite common along with idiopathic GIN in Western countries (31,32), whereas tuberculosis is the most common cause of GIN in endemic areas such as India (12,13).

The number of studies on granuloma/granulomata formation in kidney is quite limited. Despite the endemicity of tuberculosis, there is no data on the prevalence of GIN in Turkey. We therefore aimed to investigate the causes of granuloma formation in the kidney and analyze long-term outcomes of these patients.

## MATERIALS and METHODS

Medical records of adults (≥18 years of age) who underwent a kidney biopsy procedure at Dokuz Eylul University Hospital between January 2000 through June 2019 were reviewed. Kidney biopsies of adult patients (≥18 years of age) with tissue sufficient to make the diagnosis were included in the study. Patients who had granuloma/granulomata formation in kidney biopsy specimens were determined. Given the numerous etiological factors for granuloma/granulomata formation, all subjects underwent a detailed evaluation in order to determine the cause. The following data were recorded: demographic details, comorbid diseases, drug exposures, clinical findings at the time of presentation, urinalysis, full blood count, serum biochemistry (creatinine, calcium, albumin, liver function tests), autoantibodies (antinuclear antibody [ANA], anti-neutrophil cytoplasmic antibody [ANCA]), complement C3 and C4, viral serology tests (hepatitis B, hepatitis C, human immunodeficiency virus), ultrasonography of the abdomen, and chest x-ray. The following work-up was carried out patients with no apparent cause for granuloma formation (excluding drug exposures): angiotensin-converting enzyme (ACE) levels, cytomegalovirus and Epstein-Barr virus serologies, acid fast bacilli in the urine, polymerase chain reaction and culture of mycobacterium tuberculosis on bronchoalveolar lavage specimens, and computed tomography of the chest and abdomen.

The date of kidney biopsy was recorded as the baseline for laboratory records. Acute kidney injury was defined according to consensus criteria (33). Rapidly progressive glomerulonephritis was defined as the loss of kidney function within days to weeks along with remarkable findings in urinalysis (34). Microscopic hematuria was accepted if >3 red blood cells per high power field were seen in urine microscopy (35). End-stage kidney disease (ESKD) was defined according to consensus criteria (36). Given that the majority of cases presented with a rise in serum creatinine, the definition of response to therapy was made as follows. Complete response was a return of serum creatinine to <0.35 mg/dL above the baseline value and partial response was a return of serum creatinine to >.0.35 mg/dL but less than twice the baseline value (37).

### Renal Histopathology

Histological data was retrieved from pathology reports. Renal biopsy specimens were evaluated using hematoxylin–eosin, Masson’s trichrome, periodic acid schiff, and methenamine silver stained sections by light microscopy. Immunofluorescent analysis was made after staining for antibodies against immunoglobulins G-A-M, complement components C3 and C1q, and kappa and lambda light-chains for immunofluorescence. Electron microscopic evaluation was not routinely performed. Previous studies used the term GIN if at least 1 granuloma in kidney sections was found (32). For this study, we have used the term ‘’granuloma/granulomata formation’’ since there is no consensus for the definition of GIN. Given that tuberculosis is endemic in our country, Ziehl-Neelsen staining was performed on kidney sections of patients with a history of pulmonary tuberculosis, on allograft kidney with granulomatous inflammation, and on patients with no identified cause of granuloma formation.

### Statistical Analysis

Quantitative variables were expressed as median with the range (minimum-maximum). Qualitative variables were expressed as proportions. Overall renal survival was estimated using the Kaplan-Meier method. Statistical analysis was performed using SPSS 22.0 version (IBM SPSS, Chicago, IL).

## RESULTS

Granuloma was identified in 19 of 1121 (1.7%) kidney biopsies performed in our hospital between 2007-2019. The median age at the time of kidney biopsy was 60 (range, 20-84), and 12 (63.2%) were male. Two of the biopsies were allograft kidney biopsies and seventeen were native kidney biopsies.

### Native Kidney Granuloma/Granulomata

Of the 17 native kidney granulomata, 11 (64.7%) were male and the median age was 60 (range, 21-84). At baseline, hypertension and diabetes mellitus constituted 52.9% (9 patients) and 29.4% (5 patients) of the cohort. Indications for kidney biopsy included acute kidney injury (AKI) in 12 (70.6%), rapidly progressive glomerulonephritis (RPGN) in 4 (23.5%), and asymptomatic urinary abnormalities in 1 (5.9%) case. The following etiologies were captured after the detailed evaluation: pauci-immune vasculitis (n=11, 64.7%), tuberculosis (n=2, 11.8%), drug-induced (n=2, 11.8%), tubulointerstitial nephritis/uveitis (TINU) syndrome (n=1, 5.9%), and systemic-lupus erythematosus (n=1, 5.9%). Detailed description of each native kidney granuloma is given in Table 1.

**Table 1:** Characteristics, treatments, and outcomes of patients with granulomatous inflammation of the native kidney.

**Age/Gender**	**Clinical presentation**	**Histology**	**Crescent**	**Diagnosis**	**Extrarenal involvement(s)**	**Treatment**	**Outcome**
84/M	RPGN, hemoptysis, hematuria, SCr:9.1 mg/dl, MPO-ANCA+	Necrotizing granulomatous inflammation	Yes	ANCA-associated GN	Lung	Corticosteroids, cyclophosphamide	ESKD
72/F	AKI, weight loss, proteinuria, SCr:2.8mg/dl	Non-necrotizing granulomatous inflammation	No	Drug-induced GIN (penicillin)	None	Corticosteroids	CR
74/M	AKI, weight loss, hematuria, hepatosplenomegaly, SCr:2,3mg/dL. ANCA-neg	Severe necrotizing granulomatous inflammation. Ziehl-Neelsen (+)	Yes	Miliary tuberculosis	Bone marrow	Ethambutol-Pyrazinamide-Isoniazid-Rifampin	ESKD
69/M	AKI, weight loss, fever, SCr:2,6 mg/dl, MPO-ANCA+	Severe neutrophilic necrotizing granulomatous inflammation	Yes	ANCA-associated GN	Liver	Corticosteroids, cyclophosphamide, AZA	PR
21/M	AKI, hair loss, myalgia, uveitis, oral aphthae, proteinuria, SCr:2.65 mg/dl	Severe non-necrotizing granulomatous inflammation	Yes	TINU syndrome	Eye, skin	Corticosteroids, cyclophosphamide, RTX, MMF, IVIG	ESKD
62/M	AKI, weight loss, fatigue, SCr:3.74 mg/dL. ANCA-neg	Severe necrotizing granulomatous inflammation	Yes	Drug-induced (non-steroid anti-inflammatory drug)	None	Corticosteroids, cyclophosphamide	ESKD
67/F	AKI, hemoptysis, SCr:1.69 mg/dl, PR3-ANCA+	Necrotizing granulomatous inflammation	Yes	ANCA-associated GN	Lung	Corticosteroids, cyclophosphamide, AZA	CR
64/M	RPGN, fever, hematuria, proteinuria, SCr:8.11 mg/dl, PR3-ANCA+	Non-necrotizing granulomatous inflammation	Yes	ANCA-associated GN	Lung	Corticosteroids, PLEX, cyclophosphamide	ESKD
60/M	AKI, dyspnea, hematuria, SCr:9.87mg/dl, MPO-ANCA+	Non-necrotizing granulomatous inflammation	Yes	ANCA-associated GN	None	Corticosteroids, cyclophosphamide, PLEX, AZA	ESKD
59/F	RPGN, fatigue, fever, hematuria, SCr:6.46mg/dl, MPO-ANCA+	Necrotizing granulomatous inflammation	Yes	ANCA-associated GN	None	Corticosteroids, cyclophosphamide, PLEX, AZA	ESKD
80/F	RPGN, fatigue, hematuria, SCr:4.61mg/dl, MPO-ANCA+	Necrotizing granulomatous interstitial inflammation	Yes	ANCA-associated GN	None	Corticosteroids, cyclophosphamide	ESKD
49/F	RPGN, weight loss, fatigue, proteinuria, SCr:4.3 mg/dl, MPO-ANCA+	Necrotizing granulomatous inflammation	Yes	ANCA-associated GN	None	Corticosteroids, cyclophosphamide, PLEX, MMF	ESKD
50/F	AKI, fatigue, weight loss, proteinuria, SCr:2.61mg/dl. ANCA-neg. ANA and Anti-dsDNA positive.	Severe necrotizing granulomatous inflammation	Yes	Systemic lupus erythematosus	Joints, skin	Corticosteroids, cyclophosphamide, PLEX	PR
52/M	AKI, arthralgia, hematuria, SCr :1.05 mg/dl, PR3-ANCA+	Non-necrotizing granulomatous inflammation	Yes	ANCA-associated GN	None	Corticosteroids, cyclophosphamide, AZA	CR
28/M	Microscopic hematuria, proteinuria, PR3-ANCA+	Necrotizing granulomatous inflammation	Yes	ANCA-associated GN	None	ACE-inhibitor	Stable
50/M	AKI, proteinuria, SCr :3.32mg/dl	Necrotizing granulomatous inflammation	No	Tuberculosis	Lung	Ethambutol-Pyrazinamide-Isoniazid-Rifampin	ESKD
60/M	RPGN, SCr 3.87 mg/dl, PR3-ANCA+	Necrotizing granulomatous inflammation	Yes	ANCA-associated GN	Lung	Corticosteroids, PLEX, cyclophosphamide	PR

**AKI: **Acute kidney injury, **AZA: **Azathioprine, **CR:** Complete remission, **ESKD: **End-stage kidney disease, **GN:** Glomerulonephritis, **MMF:** Mycophenolate mofetil, **NR:** No response, **PLEX:** Plasma exchange, **PR:** Partial remission, **RPGN: **Rapidly progressive glomerulonephritis, **RTX:** Rituximab, **SCr:** Serum creatinine.

### Pauci-Immune Vasculitis

**Figure 1:**
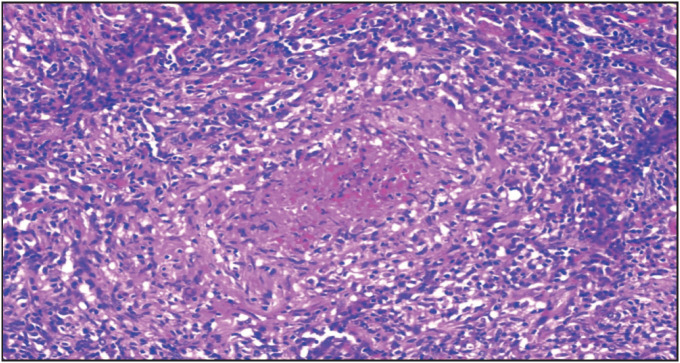
Necrotizing granulomatous interstitial inflammation in a case with granulomatous microscopic polyangiitis (H&E, x40).

The median age of these patients was 60 (range, 28-84), and 7 (63.6%) were male. Clinical presentations were as follows: AKI (6 patients, 54.5%), RPGN (4 patients, 30.8%), and asymptomatic urinary abnormalities (1 patient, 7.7%). The median serum creatinine at baseline was 4.46 (range, 1.69-9.87) mg/dL. Six patients required hemodialysis at the time of diagnosis. Seven of these had positive antibodies against myeloperoxidase (MPO-ANCA), and 4 had positive antibodies against proteinase-3 (PR3-ANCA). All of these patients were pauci-immune based on immunofluorescence microscopy findings. Lung involvement was evident in 5 (38.5%) of patients. Histology was remarkable for severe necrotizing granulomatous inflammation with crescent formation in the majority (Figure 1). All subjects received glucocorticoids while some were also treated with a mixture of cyclophosphamide, plasma exchange, azathioprine, and/or mycophenolate mofetil. Among the 10 patients who presented with AKI or RPGN, 6 had no response, 2 had partial response, and 2 achieved a complete response. The patient who had asymptomatic urinary abnormalities did not receive immunosuppressive therapy and findings in urine persisted following therapy with angiotensin-converting enzyme inhibitor. During the median follow-up of 16 (range, 1-84) months, 6 (54.5%) patients developed ESKD and 2 (18.2%) of them died.

### Tuberculosis

Two patients had tuberculosis of the kidney. Both subjects already had a diagnosis of tuberculosis of the lung by polymerase chain reaction and sputum culture prior to kidney biopsy.

The first patient was a 74-year-old-male with long-standing hypertension. The clinical presentation was AKI, with a serum creatinine level of 2.3 mg/dL. Kidney functions deteriorated and hemodialysis was initiated. There was a history of lung tuberculosis and Ziehl-Neelsen staining was positive in the kidney. In addition to severe necrotizing granulomatous inflammation, there was also crescent formation in the histology. A subsequent bone marrow biopsy also showed severe granulomatous inflammation. The patient received a combination of rifampin, isoniazide, ethambutol, and pyrazinamide, but could not come off dialysis. Unfortunately, the patient died 3 months after the kidney biopsy.

Indication for kidney biopsy in the second patient, a 50-year-old male, was AKI, with a serum creatinine of 3.32 mg/dL. Nephrotic syndrome was also evident, with 7.9 grams/24 hours of urinary protein excretion. Kidney biopsy showed severe necrotizing granulomatous intersitial nephritis. The same treatment protocol was given for tuberculosis. Unfortunately, the patient developed ESKD within 3 months after the kidney biopsy. Although both cases had a rise in serum creatinine after hospitalization, baseline serum creatinine levels were not known. They were both HIV-negative. Kidney histology revealed moderate to severe interstitial fibrosis/tubular atrophy, suggesting a preceding chronic damage.

### Tubulointerstitial Nephritis/Uveitis (TINU) Syndrome

**Figure 2:**
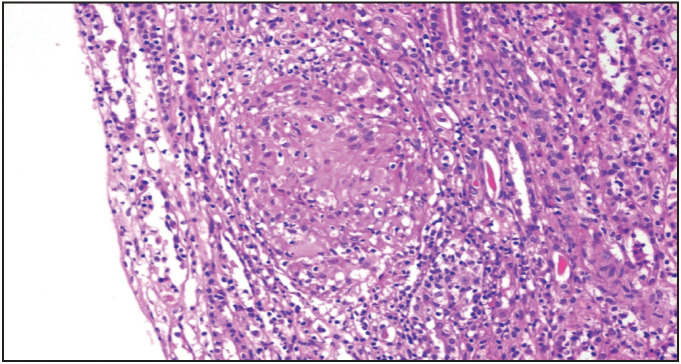
Non-necrotizing granulomatous interstitial inflamma-tion in a case with tubulointerstitial nephritis/uveitis (TINU) syndrome (H&E, x40).

The patient with TINU syndrome was a 21-year-old-male, who was admitted to the hospital for allopecia totalis, red eye, and malaise. Examination of the eye was compatible with anterior uveitis. Serum creatinine was 3.3 mg/dL and proteinuria was subnephrotic. Urine sediment showed pyuria but no microhematuria. Serological work-up was unremarkable. Kidney histology showed severe tubulointerstitial granulomatous inflammation with mild interstitial fibrosis and crescent formation. There was non-necrotizing granulomatous inflammation (Figure 2). Following treatment with cyclophosphamide and glucocorticoids, the alopecia and uveitis completely resolved with a mild improvement in kidney functions. However, kidney functions deteriorated during the following year requiring permanent dialysis, despite therapy with mycophenolate mofetil, rituximab, and intravenous immunoglobulin.

### Drug-Induced GIN

The first patient with drug-induced GIN, a 72-year-old woman, presented with AKI. Abnormal test results included a serum creatinine of 2.8 mg/dl with mild (<1 g/day) proteinuria and pyuria, but no microhematuria. Penicillin was given for upper respiratory tract infection a week before the AKI event. Kidney biopsy showed granulomatous inflammation and mild interstitial fibrosis and tubular atrophy (IFTA). Work-up for possible other etiologies such as tuberculosis, sarcoidosis, and inflammatory rheumatic diseases were unremarkable. Glucocorticoids provided a complete remission with no further relapse during the following 63 months of follow-up.

The second patient was a 62-year-old male with long-standing hypertension and diabetes. The presentation was AKI, with a serum creatinine of 3.74 mg/dL, which climbed from a baseline of 1.5 mg/dL. Urine sediment was active and the kidney biopsy showed severe necrotizing granulomatous interstitial nephritis with multiple multinucleated giant cells, and crescent formation. A detailed work-up including ANA and ANCA tests, serum calcium, hepatitis serology, ACE levels, and chest CT were unremarkable. The patient stated that he received daily non-steroid anti-inflammatory drugs within the last 2 weeks for headache. Due to crescents in histology, the patient received a combination of corticosteroids and cyclophosphamide. Unfortunately, he developed ESKD with no response to therapy during the follow-up of 6 months.

### Systemic Lupus Erythematosus

This was a 50-year-old woman with a history of chronic polyarthritis in small joints of the hand. Her presentation was with AKI, with a serum creatinine of 2.61 mg/dL. Baseline serum creatinine was told to be normal. There was approximately 3 g/24 hours of urinary protein excretion, but no hematuria. A kidney biopsy showed severe necrotizing granulomatous interstitial nephritis with crescent formation. Immunofluorescence was negative for immunoglobulins and complement. ANA and Anti-double stranded DNA (Anti-dsDNA) results were positive. Induction immunosuppression included corticosteroid and cyclophosphamide. The patient had partial response and she was maintained with azathioprine. After partial response, kidney functions remained stable with no further relapse during the follow-up of 52 months.

Among all patients with native kidney granuloma formation, the median follow-up was 24 months. Ten (58.8%) patients developed ESKD, and 4 (23.4%) died. Overall, the median estimated renal survival of 17 patients with granuloma/granulomata in the native kidney was 12 months.

### Granuloma in the Allograft Kidney

The first patient with granuloma in the allograft kidney, a 49-year-old female, had allograft dysfunction 7 years after the kidney transplantation. The primary etiology of ESKD was chronic pyelonephritis. Serum creatinine during allograft biopsy was 1.1 mg/dL, which increased from a baseline of 0.7 mg/dL. Urine tests showed a subnephrotic proteinuria and microhematuria. Biopsy showed granulomatous inflammation with crescents. There was no lung or upper respiratory-tract involvement. Detailed work-up including ANCA tests and other autoantibodies were all negative. Following glucocorticoid therapy (1 mg/kg), she achieved a complete remission with the serum creatinine returning to baseline levels around 0.7 mg/dl. Renal function remained stable for the following 68 months.

The second patient with granuloma in the allograft kidney was a 20-year-old male. He presented with a rising serum creatinine, from 1.2 mg/dL to 2.67 mg/dL. This was 11 years after the kidney transplantation and the primary etiology of the ESKD was chronic glomerulonephritis. Native kidney biopsy was compatible with immune-complex glomerulonephritis (including C1q positivity on immunofluorescence microscopy) but no granuloma, ANA and ANCA tests were negative. After a detailed evaluation, the cause of granuloma formation could not be found, and the etiology was deemed to be idiopathic. With the introduction of 1 mg/kg of glucocorticoids and continuation of mycophenolate mofetil with calcineurin-inhibitor, he achieved a complete response with a serum creatinine returning close to the baseline levels of 1.4 mg/dL.

## DISCUSSION

With this case series, we have observed that the ANCA-associated vasculitis was the most common cause of granuloma formation in the kidney. The number of patients with tuberculosis of the kidney is probably overlooked, since kidney biopsy is rarely performed in patients with tuberculosis of the lung. There were only a few patients who had acute onset disease with GIN, while the majority had more chronic onset diseases, such as chronic rheumatic conditions, vasculitis, and infections.

Previous reports included several cases with sarcoidosis of the kidney (38); yet we have not observed any, despite detailed diagnostic tests such as angiotensin-converting enzyme levels, computed tomography, and bronchoscopy were performed. Excluding 2 cases of drug-induced GIN and 2 allograft biopsies, more than half of our patients developed ESKD. This is probably be due to the high frequency of glomerulonephritis with crescents in our cohort and low number of cases with drug-induced GIN and absence of sarcoidosis, rather than granuloma formation itself. Thus, diseases causing acute granulomatous inflammation in the kidney, which may respond better to treatment were less frequently observed in our cohort. Indeed, Zajjari et al. stated that the outcome was good in patients with drug-induced GIN or sarcoidosis (39). In contrast to the outcomes of our patients, Joss et al. reported quite acceptable renal response to therapies (32). However, the authors excluded cases with crescents from their study, as these were accepted as secondary GIN. We have not excluded these subjects, since GIN frequently occurs secondary to a systemic disease such as autoimmune disorders or particular infections. Crescent formation was also evident in one of our patients with tuberculosis and the one with TINU syndrome, which would be another argument to support the inclusion of patients with crescents.

Crescent formation with granulomatous inflammation is characteristic of granulomatous polianjitis (GPA), yet granulomatous inflammation is frequently seen in lung biopsies rather than kidney specimens (40). It is not known whether renal survival is worse among subjects with GPA who have granulomatous inflammation in the kidney versus GPA without renal granulomatosis. This issue requires further study. In a multicenter study, kidney biopsies of patients with pauci-immune crescentic glomerulonephritides were classified according to the extent of the lesions in the Bowman space, and the authors used the term ‘’full moon’’ for those who had circumferential crescents (41). The main message of the paper was that patients with full moon crescents had more unfavorable renal survival. Interestingly, granuloma formation was more common in patients with full moon crescents. Among the pauci-immune glomerulonephritides, GPA and eosinophilic GPA are known to cause granulomatous inflammation. Although ANCA against proteinase (c-ANCA) is usually the positive antibody found in GPA, p-ANCA positivity was more common in this cohort.

Another typical chronic disease that may cause granulomatous inflammation in the kidney is tuberculosis. Renal tuberculosis is easily overlooked, and the diagnosis sometimes made post-mortem (42). The unfavorable outcomes of our 2 cases with renal tuberculosis may be due to the delayed diagnosis. Moreover, despite a high index of clinical suspicion, the diagnosis of GIN secondary to tuberculosis may be difficult and require PCR-based techniques (43). Microorganisms may not be detected in histological examination of the kidney. Ziehl-Neelsen staining helped only in 1 of 9 cases in the study by Agrawal and co-workers (43). The Ziehl-Neelsen stain result was positive in 1 of 2 our cases with tuberculosis. Some authors recommend combining the auramine O stain in order to increase sensitivity and specificity for the detection of tuberculosis (44). Timely diagnosis and early treatment of tuberculosis is associated with more favorable outcomes (42). GIN due to tuberculosis is even more common among subjects infected with HIV, and is associated with increased mortality in that case (45). None of our cases were HIV-positive. Similar to our paper, tuberculosis was not the predominant etiology in some of the previous reports from endemic locations (46).

One chronic systemic inflammatory disease causing GIN is the TINU syndrome. The presentation as GIN is quite rare for this syndrome (30). The presence of a crescent in our patient with TINU is also unusual. Another unexpected thing in our case is the unfavorable outcome, despite the prescription of a mixture of immunosuppressive drugs including glucocorticoids, cyclophosphamide, and rituximab. The patient uneventfully developed ESKD and was maintained on hemodialysis. Our case is the exception rather than the rule, and more studies are needed to delineate the prognostic impact of TINU syndrome on kidney outcomes.

Similar to native kidney GIN, the etiological factors are numerous in allograft kidney GIN, including several acute and chronic disorders. Infections are the most common cause of GIN in allograft kidneys (47). For transplant patients, it is important to determine if the etiology of ESKD recurs after kidney transplantation. Data for transplant kidney GIN are more lacking and come from case reports (Table 2). It is not clear if GIN recurs after kidney transplantation. However, recurrences of sarcoidosis (48), idiopathic GIN (49), TINU (50,51), and crescentic GN (47) were reported. The potential to recur probably depends on the etiology but the majority of case reports and the data from our study indicate a favorable survival in most patients, although some developed graft loss. Concurrent rejection episodes may occur and contribute to graft failure in some patients with GIN (52).

**Table 2:** Reports of Granulomatous Interstitial Nephritis in Allograft Kidneys.

**Study**	**n**	**Time after KTx (mean)**	**Etiology**	**Recurrence**	**Outcome**
Alsaad et al.(53)	1	12 years	Adenovirus	No	Recovered
al Sulaiman et al. (54)	2	~2 years	Tbc	No	Graft loss
Asim et al.(55)	1	31 days	Adenovirus	No	Recovered
Aouizerate et al. (56)	5	Median 12 months	Sarcoidosis	Yes	1 death, 1 stable, 1 improved
Baden et al.(57)	1	9 years	Coccidioidomycosis	No	Dead
Bagnasco et al. (58)	1	12 days	Candida	Donor-transmitted	Recovered
Barraclough et al. (59)	1	14 days	Adenovirus	No	Improvement
Bijol et al. (31)	3	3 weeks	Bactrim (1), Unknown (2)	N/A	N/A
Brown et al.(60)	1	1 year	Sarcoidosis	Yes	Recovered
Farris et al.(47)	22	Mean 552 (range, 10-5898) days	Viral (5), bacterial (5), drugs (5), Idiopathic (4), fungal (2), GPA (1)	1 (GPA)	22.2% graft loss due to infections, others improved/recovered
Gaspert et al.(61)	1	2 months	Adenovirus	No	Improvement
Gonçalves et al. (62)	3	N/A	Tbc	N/A	N/A
Hatlen et al.(63)	1	6 weeks	Adenovirus	Donor-transmitted	Recovered
Hotta et al.(64)	3	6 (3-15) months	Idiopathic (2), Drug (1)	No	All recovered
Josephson et al. (65)	2	24 months	Drug	No	Graft-loss due to rejection
Khaira et al.(52)	3	Range, 3-13 years	Tbc	?*	Graft loss (1), stable/improved (2)
Kukura et al.(66)	1	3 years	Sarcoidosis	Yes	Stable
Lachiewicz et al. (67)	1	20 months	Adenovirus	No	No improvement
Lapasia et al.(68)	3	Median 4 (range, 1-6) weeks	Infection (1), Drug (1), Idiopathic (1)	No	Improved (Infection, Drug) Graft loss (Idiopathic)
Lorimer et al.(69)	4	Range, 12-26 months	Tbc	No	Graft loss (2) Improved (2)
Meehan et al.(70)	3	Median 7 (range, 1-24) months	Tbc (1), Candida (1) E. coli /antibiotics (1)	No	1 death (Candida), 1 stable (Tbc), 1 transplant nephrectomy (E.coli)
Ozdemir et al. (71)	3	7.3±4.6 months	Tbc (2), Candida albicans (1)	No	Graft loss
Parasuraman et al. (72)	1	24 months	Adenovirus	No	Recovered
Park et al. (73)	1	10 months	Adenovirus	No	Improved
Shen et al.(74)	1	6 years	Sarcoidosis	Yes	Recovered
Storsley and Gibson (75)	1	6 weeks	Adenovirus	No	Improved
Sujeet et al.(76)	1	3 years	Adenovirus	No	Recovered
Teranishi et al. (49)	1	8 months	Idiopathic	Yes	Recovered
Tse et al. (77)	1	9 months	Rhodococcus	No	Stabled
Varma et al.(78)	1	14 days	Adenovirus	No	Recovered
Vargas et al.(48) (pediatric)	1	2 years	Sarcoidosis	Yes	Died of disseminated histoplasmosis
Veer et al. (79)	1	1 year	Adenovirus	No	Recovered
Zhang et al.(80)	3	6 (range, 4-12) months	BKV-associated GIN (3)	No	Recovered

*Etiology of ESKD was unknown in 1 case. **CR: **Complete remission, **GPA:** Granulomatous polyangiitis, **N/A:** Not available, **PR:** Partial remission, **Tbc:** Tuberculosis

Our study contains a number of limitations. This was a descriptive study with a small sample size, and etiologies of renal granuloma were determined retrospectively. In the majority of our cases, GIN was probably caused by chronic kidney damage, and it may not be reasonable to compare outcomes of acute causes of GIN versus those with subacute/chronic damage. Despite these significant limitations however, there are only a few studies on GIN and our data shows the considerably unfavorable renal survival of patients, unlike previous case series (Table 3). This is particularly the case for patients with chronic causes GIN, such as GPA. All of our cases had granuloma/granulomata formation but it is not clear if all of them should be regarded as GIN. There is no consensus for the definition of GIN, and this issue should be studied. Effects of granuloma formation on kidney outcomes may not be the same for each etiology, and it may not be straightforward to use a common definition for all. Apparently, acute and chronic causes of GIN should be separately evaluated.

**Table 3:** Previous Studies on Native Kidney Granulomatous Interstitial Nephritis.

**Study**	**n**	**Era**	**Male gender**	**Age, mean**	**SCr, mean**	**Etiology**	**Renal recovery**
Agrawal et al. (43)	17	2004-2014	64.7%	35	6 ±2.3 (tbc) 4.8±1.5 (sarcoid) 2.2±1.3 (idiopathic)	Tbc (52.9%), Idiopathic (23.5%), Sarcoidosis (17.6%), Fungal (5.9%)	The majority responded to therapy. Two dialysis-dependent (Tbc and sarcoidosis), 1 mortality (tbc)
Bijol et al.(31)	35 (0.5% of all biopsies)	1987-2004	50%	52 (range, 21-84)	4.1 (range, 1.5-12.8)	Sarcoidosis (28.9%), Drug (44.7%) GPA (15.9%)	N/A
Gupta et al. (12)	16 (1.08% of all biopsies)	2009-2013	62.5%	34 (range, 12-68)	6.25 ± 3.53	Tbc (56.3%), Cresc. GN (12.5%) Idiopathic (12.5%), Drugs (12.5%) Infection (6.2%)	The majority responded to therapy. Two dialysis-dependent (Tbc), 1 underwent transplantation (Tbc)
Javaud et al. (81)	40 (1.37% of all biopsies)	1991-2004	62.5%	53	median GFR 26 mL/min (range, 5-80)	Sarcoidosis (50%), Drug (17.5%) Tbc (7.5%), GPA (5%), Leprosy (2.5%), M. avium (2.5%), Crohn (2.5%)	3 dialysis-dependent (GPA, Crohn, drug-induced), 1 transplant (sarcoidosis), 2 mortality (drug-induced GIN, Tbc)
Joss et al.(32)	18	1990-2004	61%	55	4.21 (1.15 to 15.41)	Idiopathic (50%), Sarcoidosis (28%), Drug (11%), TINU (11%)	None required long-term renal replacement therapy
Karmakar et al. (46)	6	N/A	33.3%	range, 14-65	range, 0.9 - 7.13	SLE (33.3%), Cresc. GN (33.3%) Idiopathic (33.3%)	N/A
Mignon et al. (82)	32 (0.9% of all biopsies)	N/A	N/A	20-76	N/A	Drug (31.2%), GPA (25%), Idiopathic (25%), Sarcoidosis (9.3%), Tbc (9.3%)	Most recovered or stable, 5 died, 1 required long-term dialysis.
Naidu et al. (83)	14 (0.5% of all biopsies)	2000 to 2012	57.1%	35 (range, 20-70)	6.7±3.8 (2.3-14.7)	Tbc (64.3%), Drug (14.4%), SLE (7.1%), GPA (7.1%), IgAN (7.1%)	5 dialysis, 1 transplant, 8 recovered/improved
Oliveira et al. (29)	21	2000-2012	57%	53 (range, 19-73)	GFR, range, 11-113 ml/min	Sarcoidosis (62%), Tbc (24%), Idiopathic (10%), Drugs (5%)	1 death (idiopathic), 1 dialysis (tbc)
Viero and Cavallo (84)	12 (5.9% of all biopsies)	1974-1994	33.3%	46 (range, 24-78)	5.1 (range, 1.9-8.7)	Drugs (25%), Sarcoidosis (25%), Infections (25%), Oxalosis (8%), GPA (8%), Idiopathic (8%)	The majority were lost to follow-up. One mortality (infection). Three developed chronic renal failure.
Zajjari et al. (39)	11 (2.7% of all biopsies)		36.4%	44.2	3.91 ± 2.07	Sarcoidosis (45.4%), Drugs (27.2%)	Patients with drug-induced GIN and sarcoidosis recovered, but no renal recovery in other etiologies

**Cresc. GN:** Crescentic glomerulonephritis, **GPA:** Granulomatous polyangiitis, **IgAN:** IgA nephropathy, **N/A:** Not available, **Tbc:** Tuberculosis.

In conclusion, ANCA-associated vasculitis appeared to be the most common cause of granuloma formation in the kidney in our study. Renal survival is significantly shortened and multicenter studies are needed in order to delineate the nature of different etiologies of granuloma formation in the kidney, and determine the best treatment option for each category.

## Conflict of Interest

The authors declare no conflict of interest.

## Ethics approval

This study was approved by the Ethics Committee of Dokuz Eylül University School of Medicine (IRB code: 2019/17-230307).

## Informed consent

Informed consent was waived due to the retrospective design, confidentiality of patient identity, and absence of any invasive procedures.
